# Effective Interventions in Restoring Mobility and Relieving Pain After Lower Limb Amputation

**DOI:** 10.26502/josm.511500282

**Published:** 2026-09-07

**Authors:** Suyeon (Su) Kim, Grace Kim, Dominick Shoha, Marcel P. Fraix, Devendra K. Agrawal

**Affiliations:** 1 Department of Translational Research, College of Osteopathic Medicine of the Pacific, Western University of Health Sciences, Pomona, California 91766, USA; 2 Department of Physical Medicine and Rehabilitation, College of Osteopathic Medicine of the Pacific, Western University of Health Sciences, Pomona, California 91766, USA

**Keywords:** Amputation, Augmented reality rehabilitation, Lower limb loss, Movement, Neuromodulation, Neuroprosthetics, Neurorehabilitation, Physical therapy, Prosthetics, Regeneration, Rehabilitation, Transfemoral amputation, Transtibial amputation, Virtual reality rehabilitation

## Abstract

Lower-limb amputation is associated with substantial functional impairment, chronic pain, and reduced quality of life, posing significant challenges for long-term rehabilitation and community reintegration. Despite advances in surgical techniques and prosthetic technologies, many individuals continue to experience limitations in mobility and persistent pain after amputation. This narrative review synthesizes current evidence on the functional consequences of limb loss and the most effective strategies for restoring mobility and managing pain following lower-limb amputation. Key approaches to rehabilitation include structured physical therapy, prosthetic optimization, and multidisciplinary care models that address biomechanical and psychosocial factors, including recovery. Pharmacologic regimens, neuromodulation, mirror therapy, and emerging surgical interventions, such as targeted muscle reinnervation, have demonstrated promising outcomes in reducing phantom limb pain and improving functional control. In addition, innovations in neuroprosthetics, wearable sensors, and data-driven rehabilitation are expanding opportunities for personalized treatments and improved prosthetic integration. However, disparities in access to rehabilitation services and advanced prosthetic technologies remain significant barriers to many patients. Continued research emphasizing standardized outcome measures, long-term clinical trials, and multidisciplinary care frameworks will be essential to improving functional outcomes and quality of life for individuals living with limb loss.

## 1. Introduction

Amputation may involve the upper or lower limb, and both forms can substantially affect independence, psychological well-being, social participation, and quality of life. As of 2017, more than 57 million people worldwide were estimated to be living with limb loss, and in the United States alone, approximately 185,000 amputations are performed each year [1,2]. Nearly 80% of these procedures are related to vascular disease and diabetes, while about 16% result from traumatic injury [3,4]. Furthermore, a 2023 global meta-analysis reported that among individuals with diabetes, the annual incidence of lower-limb amputation ranges from 95 to 140 per 100,000 people [5–10]. Collectively, these findings highlight limb loss as a significant public health concern with substantial consequences for mobility, independence, and quality of life.

Upper-limb amputation primarily compromises reach, grasp, bimanual activities, self-care, work, and community participation, whereas lower-limb amputation most directly restricts standing, transfers, gait, balance, endurance, and community mobility [11,12]. Because the evidence synthesized in this review predominantly concerns lower-limb loss, the discussion that follows focuses on lower-limb amputation while recognizing the distinct functional burden of upper-limb amputation.

The diverse etiologies of limb loss underscore the complexity of addressing patients’ clinical, rehabilitative, and psychosocial needs. While there have been advances in surgical techniques, prosthetic technology, and rehabilitative care, many amputees continue to endure functional limitations such as impaired gait and compromised balance, which may hinder return to work and community engagement [13,14]. Post-amputation pain remains highly prevalent. Phantom limb pain, residual limb pain, and secondary musculoskeletal strain, particularly in the lower back, can significantly impede recovery and contribute to emotional distress, including depression and anxiety [15–20]. In response to these challenges, healthcare providers employ a range of interventions designed to restore function and manage pain. Rehabilitative approaches such as mirror therapy and mirror visual feedback have demonstrated reductions in phantom limb pain severity [21]. Additionally, pharmacological agents, including gabapentin, morphine, and ketamine, offer short-term symptom relief [22–24]. Regional analgesic strategies, such as peripheral nerve blocks and epidural analgesia, have been shown to reduce postoperative pain and support functional gains [25–30]. Additionally, emerging innovations, including targeted muscle reinnervation and advanced prosthetic control systems, have shown promise in improving neuromuscular coordination and enabling more natural limb function [31,32].

This comprehensive literature review synthesizes current evidence on functional rehabilitation and pain management following limb amputation, highlighting established practices, ongoing debates, and key gaps. By examining established practices, emerging innovations, and persistent knowledge gaps, this work aims to identify the strategies that most effectively promote recovery, optimize long-term outcomes, and advance integrated, patient-centered approaches to care for individuals with amputations.

## 2. Impact on Mobility

### 2.1 Primary Mobility Limitations

Lower-limb amputation is associated with significant and often persistent functional limitations that affect mobility, gait, balance, and the ability to perform daily activities [33,34]. While some difficulties are temporary and arise during the immediate postoperative recovery period, many individuals face long-term challenges that persist well beyond the initial rehabilitation phase [35,36]. These limitations can significantly influence independence and overall quality of life, even in the context of modern surgical techniques, advanced prosthetic technologies, and structured rehabilitation programs. Among all functional domains, mobility loss is among the most substantial. Limb loss disrupts normal biomechanics, resulting in slower walking speeds, asymmetrical gait, and increased energy expenditure during ambulation [37]. Amputees often have trouble climbing stairs, walking on uneven terrain, and performing transitional movements such as turning or sit-to-stand [38]. Mobility challenges are particularly prominent among individuals with dysvascular lower-limb amputations [39]. Lee et al. [40] state that only about half of these individuals utilize walking as their primary mode of mobility one year after amputation, indicating substantial physical and adaptive demands of gait restoration. Reduced ambulatory capacity may lead to increased reliance on assistive devices, decreased physical activity, and heightened risk of secondary complications, including deconditioning and joint pain [41].

Balance and endurance are also frequently compromised following lower-limb loss. The absence of proprioceptive feedback and altered weight distribution increase postural instability and the risk of falls [42]. Decreased confidence in balance can perpetuate inactivity and reduced cardiovascular endurance [43]. Lower-limb amputees often demonstrate shorter walking distances and reduced exercise tolerance compared to able-bodied individuals [44]. Unfortunately, even with modern prosthetic designs, consequences such as fatigue, socket discomfort, and high energy expenditure limit the long-term use of prosthetics and independent ambulation [45,46].

Additionally, functional limitations may negatively impact a patient’s occupational and social domains. The time to return to work can often be prolonged due to reduced ability to stand, walk, and perform job-related physical tasks, which creates challenges. Evidence indicates that fewer than one in three individuals with dysvascular lower-limb amputation successfully return to employment [40]. Beyond work productivity, limited mobility and prosthetic discomfort may restrict participation in community activities, hinder social engagement, and contribute to psychological distress such as anxiety, depression, and reduced self-efficacy [47].

### 2.2 Early Mobility Loss Following Lower-Limb Amputation

In a Danish prospective cohort study, Madsen et al. [48] examined short-term functional outcomes following dysvascular lower-limb amputation using the Barthel Index (BI) to measure changes in activities of daily living (ADL) from pre-amputation to Day 21 post-surgery, reporting a significant decline across all ten BI domains [48]. These domains included personal hygiene, bathing, eating, toileting, dressing, bowel and bladder control, ambulation or wheelchair use (if non-ambulatory), bed-chair transfers, and stair climbing [49]. The study identified older age (≥ 65 years), higher ASA score, and lack of prosthesis assessment before discharge as significant predictors of continued dependence [50]. Similarly, in Italy, Brunelli et al. [51] evaluated 135 post-amputation patients and found that although BI scores improved during rehabilitation, the tool underestimated mobility changes, those related to transfers and prosthetic adaptation [51]. Elevated plasma homocysteine (H-HCY), a marker of vascular disease, was significantly associated with lower rehabilitation effectiveness, emphasizing how metabolic and comorbid factors influence early recovery. Complementing these findings, Venkataraman et al. [52] analyzed rehabilitation outcomes among U.S. inpatients with dysvascular amputations and reported that early initiation of rehabilitation (within 7 days) led to significantly higher Functional Independence Measure (FIM) scores and greater discharge mobility compared to delayed therapy, even after adjusting for age, amputation level, and comorbidities [52]. Taken together, these studies demonstrate that early functional decline after amputation is profound and consistent across populations, but recovery potential is strongly influenced by both non-modifiable factors (age, baseline health, vascular/metabolic status) and modifiable factors (timing of rehabilitation, prosthesis evaluation, and individualized discharge planning). Collectively, this evidence underscores the importance of early, multidisciplinary rehabilitation interventions to mitigate immediate postoperative dependence and optimize mobility within the first month after surgery.

### 2.3 Transtibial Versus Transfemoral Amputation: Recovery, Prosthetics, and Rehabilitation

The distinction between transtibial and transfemoral amputation is clinically important because preservation of the knee joint changes the expected rehabilitation course, prosthetic prescription, gait demands, and likelihood of independent ambulation. Transtibial rehabilitation generally emphasizes residual limb management, prosthetic alignment, ankle-foot substitution, balance, and gait symmetry. Transfemoral rehabilitation additionally requires mastery of a prosthetic knee, greater hip strength and trunk control, more intensive fall prevention training, and accommodation of higher metabolic demands during walking [53,54].

The level of amputation is one of the most critical determinants of long-term functional outcomes. Evidence consistently shows that higher (more proximal) levels of amputation are associated with greater mobility limitations and reduced independence. In a large-scale systematic review and meta-analysis, Penn-Barwell et al. [55] demonstrated that patients with below-knee amputations (BKA) retained superior mobility compared to those with above-knee amputations (AKA) or bilateral limb loss, exhibiting greater ambulatory capacity and functional independence. These findings have been corroborated by subsequent clinical and observational studies. For instance, Kaur et al. [56] reported that individuals with BKA exhibited more symmetrical gait patterns, higher average walking speeds, and longer daily prosthesis use relative to those with AKA [56]. Similarly, Seker et al. [57] found significantly higher Amputee Mobility Predictor (AMP) scores among transtibial amputees, both with and without prostheses, suggesting better overall functional mobility [57]. These advantages are largely attributed to the preservation of the knee joint, which enables more natural limb mechanics, improved prosthetic control, and lower energy expenditure during ambulation [58,59].

In contrast, individuals with transfemoral amputations face more profound biomechanical limitations. The absence of the knee joint precludes active plantar flexion and push-off during gait, forcing patients to rely primarily on hip musculature for forward propulsion [60]. This compensatory mechanism increases energy consumption and reduces gait efficiency [61,62]. Such adaptations contribute to slower walking speeds, greater gait asymmetry, and higher physical demands during daily activities such as transfers and stair climbing. During sit-to-stand maneuvers, individuals with lower-limb amputations (LLA) tend to lean toward the intact limb, resulting in greater load and higher knee joint moments on that side [60]. Over time, this preferential loading contributes to the development of contralateral knee osteoarthritis, particularly among individuals with AKA [63]. These mechanical and metabolic inefficiencies collectively reduce endurance, functional participation, and overall quality of life.

Beyond physical impairment, amputation also imposes enduring barriers to employment and social reintegration. Reduced walking endurance, prosthetic discomfort, and secondary musculoskeletal conditions limit activity participation, while psychological distress—including depression, anxiety, and altered self-identity, further impairs rehabilitation outcomes [64]. The AHA emphasizes that consistent prosthesis use and walking ability are among the strongest predictors of quality of life and social participation following amputation [65]. To enhance these outcomes, the AHA recommends integrated behavioral health interventions during rehabilitation to facilitate psychosocial adjustment and promote community reintegration.

Physical performance measures such as the Timed Up and Go (TUG) test and self-reported mobility ratings are positively correlated with re-employment and social participation among amputees [66,67]. Conversely, persistent pain, prosthetic dissatisfaction, and limited prosthesis use are negatively associated with return-to-work outcomes [68–70]. The U.S. Department of Veterans Affairs and Department of Defense (VA/DoD) clinical practice guideline advises comprehensive rehabilitation that includes pain management, psychosocial screening, and standardized mobility assessments to mitigate these barriers and improve long-term outcomes [71,72]. Despite these recommendations, recent studies indicate that fewer than one-third of dysvascular amputees successfully return to work, with earlier data reporting re-employment rates of approximately 40% [40]. These findings underscore that successful reintegration after amputation depends not only on physical capacity but also on psychological resilience and environmental support. Thus, optimizing both functional rehabilitation and psychosocial interventions remains essential for restoring autonomy and improving quality of life in this population.

### 2.4 Determinants of Functional Recovery

Psychological and behavioral factors exert a profound influence on post-amputation recovery, shaping both functional outcomes and pain perception [73,74]. Motivation, self-efficacy, and coping style influence not only engagement in rehabilitation but also tolerance of discomfort, adaptation to prosthetic use, and sustained long-term mobility [74,75]. Individuals who approach rehabilitation with optimism and problem-solving behaviors tend to regain higher levels of independence, whereas maladaptive responses such as catastrophizing and avoidance often perpetuate pain, prosthesis abandonment, and functional decline [76]. Cognitive-behavioral therapy (CBT), motivational interviewing, and peer mentoring have each shown promise in enhancing self-efficacy and fostering active engagement in therapy [77–80]. Furthermore, virtual and augmented reality interventions are emerging as tools to promote body schema restoration, improve adherence to mirror therapy, and reduce phantom limb pain [81–83]. Targeting psychological barriers early in rehabilitation fosters resilience, a biopsychosocial multiplier that accelerates recovery, optimizes prosthetic adaptation, and sustains meaningful participation in daily life.

Beyond the psychological domain, surgical and anatomical factors lay the physiological foundation for functional recovery. Advances in operative techniques, including Targeted Muscle Reinnervation (TMR) and Regenerative Peripheral Nerve Interfaces (RPNI), have significantly reduced neuroma-related pain and enhanced the control of myoelectric prostheses [84–88]. Likewise, meticulous attention to muscle reattachment, bone length preservation, and limb contouring during amputation improves prosthetic suspension and load distribution, thereby facilitating smoother gait mechanics and endurance [12,89,90]. Emerging methods, such as osseointegration, direct skeletal anchoring of the prosthesis, restore proprioceptive feedback and reduce energy expenditure, offering a more natural and stable interface between the body and device [91–93]. In this context, a function-oriented surgical approach that anticipates prosthetic integration becomes a pivotal determinant of mobility and comfort, reinforcing that recovery begins in the operating room as much as in the rehabilitation clinic.

Functional recovery after amputation is not determined solely by clinical factors; socioeconomic and environmental contexts also play equally critical roles. Access to advanced prosthetic technologies, comprehensive rehabilitation services, and pain management interventions is often constrained by insurance coverage, geographic location, and the structure of the health system [94–97]. Individuals in rural or low-resource settings frequently experience delayed prosthetic fitting and limited follow-up care, resulting in poorer mobility and reduced participation [98–100]. Cross-national disparities further highlight how policy, infrastructure, and funding differences shape recovery trajectories, with higher-income countries demonstrating superior long-term outcomes compared to regions where prosthetic access remains scarce [101,102]. Social support and family involvement also strongly influence adaptation, motivation, and adherence to therapy, while cultural perceptions of disability can either foster empowerment or reinforce isolation [103–105]. Recognizing these social and environmental determinants is crucial to developing equitable rehabilitation models that bridge care gaps and promote functional independence across diverse populations.

## 3. Pain After Amputation

### 3.1 Types and Mechanisms of Post-Amputation Pain

Post-amputation pain (PAP) is a multifaceted clinical problem that significantly affects individuals following limb loss. It encompasses three major categories: phantom limb pain (PLP), residual limb pain (RLP), and secondary musculoskeletal pain. PLP refers to painful sensations perceived in the missing portion of the limb, often described as burning, stabbing, or cramping [106]. It is reported in up to 85% of amputees and can persist for years after surgery [107–109]. RLP, in contrast, arises from the stump tissues and is frequently linked to peripheral nerve injury, neuroma formation, ischemia, infection, or prosthetic pressure [110]. Secondary musculoskeletal pain develops due to compensatory movement patterns, joint overload, or spinal malalignment resulting from altered biomechanics [111]. Although these syndromes overlap clinically, differentiating among them is essential for accurate diagnosis and management.

The underlying mechanisms of post-amputation pain involve interacting peripheral, central, and psychosocial pathways. At the peripheral level, nerve transection leads to aberrant axonal sprouting and neuroma formation, producing ectopic discharges that amplify nociceptive input [112]. Persistent afferent firing contributes to spinal dorsal horn hyperexcitability and maladaptive neuroplasticity in supraspinal centers [113]. Functional neuroimaging studies have demonstrated that cortical reorganization within the somatosensory and motor cortices correlates with PLP intensity, implicating central sensitization and disrupted body schema. Psychological factors, including pain-related anxiety, catastrophizing, and pre-amputation pain memory, can further sustain central hyperexcitability. This biopsychosocial model underscores that post-amputation pain is not solely a peripheral phenomenon but a dynamic interplay between neural and cognitive processes.

### 3.2 Impact on Quality of Life and Rehabilitation Outcomes

Persistent pain after amputation exerts a profound impact on mobility, independence, and psychosocial well-being [114,115]. Chronic PAP often limits prosthetic tolerance, reduces walking speed, and increases energy expenditure during ambulation, leading to early fatigue and dependency on assistive devices [38,116,117]. Individuals with significant pain are less likely to achieve community ambulation or return to work, and they frequently experience greater disability scores and lower health-related quality of life indices [14–16,118]. In addition, compensatory overuse of the intact limb can result in contralateral joint degeneration and spinal discomfort, compounding overall morbidity [119,120]. Psychologically, uncontrolled pain contributes to sleep disturbance, depression, anxiety, and social isolation, all of which impede motivation for rehabilitation [121,122]. Pain-related fear and avoidance behaviors can delay prosthetic training and limit participation in therapy, leading to functional deconditioning [123–125]. The cumulative effect is a negative feedback loop in which pain reduces activity, inactivity exacerbates disability, and diminished function reinforces psychological distress [126]. Adequate pain control is therefore integral to restoring both physical function and emotional resilience, serving as a prerequisite for successful reintegration and long-term rehabilitation outcomes.

### 3.3 Current Pain Management Approaches

The management of post-amputation pain requires a multimodal and interdisciplinary strategy targeting both peripheral and central mechanisms. Pharmacologic therapies remain the first line of treatment, including anticonvulsants (gabapentin, pregabalin), antidepressants (tricyclics, SNRIs), NMDA receptor antagonists, and limited use of opioids for acute pain control [127–129]. Topical agents and local anesthetics may provide adjunctive relief for stump discomfort [130]. Surgical and interventional techniques—such as targeted muscle reinnervation (TMR), regenerative peripheral nerve interfaces (RPNI), and peripheral nerve capping- address neuroma-related pain by re-establishing physiological nerve targets [87,131,132]. Neuromodulation approaches, including spinal cord stimulation (SCS) and dorsal root ganglion (DRG) stimulation, have demonstrated efficacy for refractory PLP and RLP, particularly when conservative measures fail [133–135].

Non-pharmacologic modalities are increasingly emphasized to address central and psychosocial components. Mirror therapy, graded motor imagery, virtual reality training, and sensory discrimination therapy modulate cortical reorganization and restore body representation [136,137]. Cognitive-behavioral therapy (CBT) and mindfulness-based interventions mitigate catastrophizing and improve coping strategies [78–80,138]. Comprehensive rehabilitation programs integrating medical, psychological, and functional interventions achieve superior outcomes compared to unimodal treatments [139,140]. The evolving paradigm in post-amputation care emphasizes personalized, mechanism-informed, and multidisciplinary management to achieve durable pain relief and enhance overall function.

## 4. Emerging and Advanced Interventions

### 4.1 Technological Innovations in Prosthetics

Advances in prosthetic design have focused on closing the gap between human intention and device responsiveness. Microprocessor-controlled prostheses (such as knees and ankles) dynamically adjust resistance and deliver powered assistance during ambulation, enhancing cadence adaptability, reducing metabolic energy expenditure, and improving safety on varied terrains [141]. These devices have demonstrated meaningful improvements in gait symmetry, walking velocity, and fall reduction in clinical trials [142,143].

Osseointegration (OI) represents another transformative innovation, providing direct skeletal attachment of the prosthesis without the need for a socket [144]. This technique improves comfort, proprioception (“osseoperception”), and range of motion, while reducing soft-tissue irritation [145]. However, complications such as infection and periprosthetic fracture remain concerns requiring long-term surveillance [146,147].

Additionally, advanced neural control systems, including targeted muscle reinnervation (TMR), regenerative peripheral nerve interfaces (RPNIs), and pattern recognition myoelectric control, allow users to command multiple prosthetic degrees of freedom using natural motor signals [148]. Integration with sensory feedback systems (vibrotactile or electrotactile stimulation) enhances embodiment and postural stability [149]. Emerging socket technologies, utilizing additive manufacturing and embedded pressure sensors, further individualize fit and enhance residual limb health [150]. Collectively, these innovations demonstrate measurable functional and quality-of-life benefits but remain limited by device cost, maintenance demands, and training requirements.

### 4.2 Neuromodulation and Neurorehabilitation

Pain after amputation remains a significant barrier to recovery, with neuropathic and central sensitization mechanisms contributing to residual and phantom limb pain. Neuromodulatory interventions, including peripheral nerve stimulation (PNS), dorsal root ganglion (DRG) stimulation, and spinal cord stimulation (SCS), offer promising pain reduction for patients who are refractory to conventional therapies [151–153]. These techniques modulate abnormal nociceptive signaling, resulting in significant improvements in pain scores, sleep quality, and functional engagement [154–157].

Noninvasive brain stimulation methods, such as repetitive transcranial magnetic stimulation (rTMS) and transcranial direct current stimulation (tDCS), have demonstrated short-term analgesic effects and benefits in cortical remapping in small clinical trials [158–160]. Surgical neurorehabilitation strategies, including TMR and RPNI, also address neuroma pain while enhancing prosthetic control by reassigning severed nerves to new motor targets [84,87,161]. When integrated with task-specific therapy and graded motor imagery or mirror therapy, these approaches facilitate cortical normalization and reduce phantom limb phenomena [162–164].

Although early evidence suggests durable pain relief and functional gains, heterogeneity in patient selection and trial design limits the generalizability of these findings [165–167]. Ongoing multicenter trials aim to clarify optimal stimulation targets, protocols, and long-term cost-effectiveness [167–169].

### 4.3 Regenerative and Biologic Approaches

Regenerative medicine has introduced biologically based solutions to improve residual limb integrity and mitigate neuropathic complications [170,171]. Surgical nerve management techniques—particularly TMR and RPNI—are supported by growing clinical evidence demonstrating substantial reductions in neuroma and phantom limb pain, along with improved myoelectric control [84,172–175]. Complementary strategies include the use of nerve conduits, biologic wraps, and extracellular matrix (ECM) scaffolds to guide axonal regeneration and modulate local inflammation [176–180].

Additionally, autologous biologics, such as platelet-rich plasma (PRP) and fat grafting, are being investigated for restoring soft-tissue volume and reducing stump pain [181,182]. Within the context of osseointegration, bioactive surface coatings and antimicrobial modifications aim to enhance bone-implant integration and reduce the risk of infection [183–188]. While these regenerative modalities are conceptually promising, robust randomized trials and standardized outcome measures are needed to validate efficacy and safety [189–193].

### 4.4 Virtual and Augmented Reality Rehabilitation

Virtual reality and augmented reality (VR/AR) platforms are emerging as novel tools for motor retraining and pain modulation [81,194]. VR-based rehabilitation programs simulate variable environments to improve gait adaptability, balance, and confidence while providing quantitative kinematic feedback [194–196]. Embodiment-focused applications allow amputees to visualize a virtual limb responding to motor or EMG input, facilitating cortical reorganization and reducing phantom pain through visual-sensory coupling [197–199].

Augmented reality systems provide real-time visual overlays to guide prosthetic alignment, foot placement, and weight-shifting exercises, enabling telerehabilitation and improved home training compliance [200–202]. Moreover, gamified interfaces linked to prosthetic sensors promote high-repetition, task-specific movement practice, enhancing neuromuscular coordination [203–205].

Preliminary studies have reported significant short-term improvements in both pain and functional outcomes with VR/AR-based protocols compared to conventional therapy [194,206,207]. However, the scalability of these technologies is constrained by hardware costs, motion sickness in some users, and a lack of standardized implementation across clinical settings [203,208,209].

## 5. Challenges and Future Directions

Despite significant progress in prosthetic technology, neuromodulation, and regenerative medicine, multiple challenges continue to impede optimal recovery and equitable access to care for individuals with limb loss [95,129]. Disparities in access, inconsistent research standards, and limited long-term evidence hinder the translation of these advances into tangible benefits in the real world [94,129,210]. At the same time, emerging trends in personalized, data-driven rehabilitation and multidisciplinary collaboration offer promising directions for the future of amputation care.

Access to rehabilitation and prosthetic care remains highly unequal worldwide [211]. Individuals from low-income, uninsured, or rural populations frequently encounter delays in prosthetic fitting, limited availability of specialized rehabilitation centers, and inconsistent follow-up care [98,212,213]. These inequities lead to poorer functional recovery, increased secondary complications, and reduced quality of life [94,95,214]. Insurance and reimbursement restrictions further constrain access to advanced prosthetic systems, osseointegration procedures, and long-term physical therapy [215]. Addressing these barriers will require coordinated policy reform, community-based rehabilitation networks, and broader implementation of tele-rehabilitation programs to improve continuity of care and accessibility of expert services [216].

A second challenge lies in the lack of standardized, long-term data across studies assessing prosthetic outcomes and pain management interventions [174,202]. Many investigations remain short in duration, use heterogeneous metrics, and fail to employ uniform patient-reported outcome measures (PROMs) [190,217,218]. Without consistent reporting frameworks, it is difficult to compare findings, perform meta-analyses, or identify predictors of durable functional success [219]. Establishing internationally recognized registries and adopting consensus-based core outcome sets will be crucial for generating reproducible, high-quality evidence that informs both clinical practice and policy development [220–222].

Looking forward, integrating personalized, data-driven, and multidisciplinary care models represents a crucial step in advancing amputation rehabilitation [221,223]. Wearable sensors, digital gait analytics, and machine learning algorithms now provide continuous feedback on limb loading, prosthesis performance, and energy expenditure, enabling real-time, individualized therapy adjustments [224–227]. By combining biomechanical, neurological, and psychosocial data, clinicians may soon be able to predict complications, optimize device selection, and tailor pain management strategies for each patient [228–230]. A truly multidisciplinary model, uniting surgeons, physiatrists, prosthetists, therapists, and mental health specialists, will be critical to achieving holistic recovery that addresses both physical and psychosocial dimensions of adaptation [223,231,232].

Future research should prioritize the continued development of neuroprosthetics that enable intuitive, bidirectional communication between the nervous system and prosthetic devices, thereby improving motor control, sensory feedback, and embodiment [233–237]. Simultaneously, greater emphasis must be placed on psychosocial resilience, including strategies to enhance coping mechanisms, social reintegration, and long-term mental health after limb loss [238,239]. Finally, establishing standardized outcome measures and conducting multicenter, longitudinal trials will provide the robust evidence necessary to evaluate safety, cost-effectiveness, and long-term durability of new interventions [190,192,240,241].

## 6. Conclusion

Lower-limb amputation remains a life-altering condition that significantly impacts mobility, independence, and quality of life. Successful recovery is shaped by a complex combination of biomechanical, neurological, psychological, and socioeconomic factors. Functional outcomes depend not only on surgical technique and amputation level, but also on timely rehabilitation, access to prosthetics, and patient engagement. Post-amputation pain remains a major barrier to recovery, requiring a multidisciplinary mechanistic approach to management. Advances in surgical techniques, prosthetic technologies, and rehabilitation strategies offer promising improvements in function and pain control, but long-term effectiveness and accessibility remain limited. Persistent disparities in care highlight the need for more equitable access, standardized outcome measures, and long-term research. Ultimately, optimizing outcomes after amputation requires an integrated, patience-centered approach that addresses both physical and psychosocial dimensions of recovery.
